# Application of Iron Magnetic Nanoparticles in Protein Immobilization

**DOI:** 10.3390/molecules190811465

**Published:** 2014-08-04

**Authors:** Jiakun Xu, Jingjing Sun, Yuejun Wang, Jun Sheng, Fang Wang, Mi Sun

**Affiliations:** Key Laboratory of Sustainable Development of Marine Fisheries, Ministry of Agriculture, Yellow Sea Fisheries Research Institute, Chinese Academy of Fishery Sciences, Qingdao 266071, China; E-Mails: chenfeng858652@163.com (J.X.); jingjingsuna@163.com (J.S.); shengjun@ysfri.ac.cn (J.S.); wendywf2002@163.com (F.W.)

**Keywords:** iron magnetic nanoparticles, surface modification, protein immobilization, application

## Abstract

Due to their properties such as superparamagnetism, high surface area, large surface-to-volume ratio, easy separation under external magnetic fields, iron magnetic nanoparticles have attracted much attention in the past few decades. Various modification methods have been developed to produce biocompatible magnetic nanoparticles for protein immobilization. This review provides an updated and integrated focus on the fabrication and characterization of suitable magnetic iron nanoparticle-based nano-active materials for protein immobilization.

## 1. Introduction

Enzyme-catalyzed reactions usually take place under relatively mild conditions, which make them ideal alternatives to various traditional chemical reactions. However, free enzymes usually have poor stability towards pH, heat or other factors and are difficult to recover and reuse [1,2]. Therefore, there is a great demand for ways to improve enzyme stability and reusability.

Immobilization techniques, as a very powerful tool, have been intensively utilized to prepare various high-performance and economically-feasible biocatalysts with improved stability and resuability. By tuning the immobilization strategies and carefully selecting the materials, the immobilized enzymes can work in a much broader pH and temperature range and show higher thermal stability than the native ones, which could be attributed to multipoint, multi-subunit immobilization or generation of favorable environments. Several new types of carriers and technologies on immobilization have been developed in the recent past to enhance the loading, activity and stability of enzymes [3,4,5,6,7,8,9,10], which decrease the enzyme biocatalyst cost for industrial application. These include cross-linked enzyme aggregates [11,12,13], microwave-assisted immobilization [14], click chemistry technology [15], mesoporous supports [16] and most recently nanoparticle-based immobilization of enzymes [17,18,19].

Magnetic metal nanoparticles have been used in protein/enzyme immobilization owing to their unique properties such as superparamagnetism, high surface area, large surface-to-volume ratio, easy separation under external magnetic fields [20,21,22,23,24]. Another key factor to take full advantages of nanoparticles such as magnetic nanoparticles is how to ideally regulate the orientation of the proteins/enzymes on the supports. Compared to porous supports, such non-porous nanoparticles have no external diffusion problems, making them more competitive especially for large scale industrial usage in solid–liquid systems (e.g., precipitated protein). However, as drawbacks compared to porous supports, proteins/enzymes immobilized on non-porous nanoparticles may suffer inactivation for soluble proteins/enzymes, especially through interaction with gas bubbles generated by strong stirring or bubbling of oxygen [25]. Such inactivation by interfaces might proceed and finally result in the irreversible activity loss due to the sustained effects such as destabilization of electrostatic, hydrophobic and hydrogen bonds [26]. The frequently utilized magnetic nanoparticles are iron oxides, among which superparamagnetic Fe_3_O_4_ nanoparticles are the most prevalent materials because they have low toxicity, good biocompatibility [27,28,29,30,31]. Since the bare iron magnetic nanoparticles often have high reactivity and easily undergo degradation upon direct exposing to certain environment, leading to poor stability and dispersity [32,33]. Various modification methods have been developed to get the soluble and biocompatible iron magnetic nanoparticles for protein immobilization [34]. This review provides an updated and integrated focus on the fabrication and characterization of suitable iron magnetic nanoparticles for protein immobilization. In addition, the applications of immobilized enzymes based on iron magnetic nanoparticles in certain fields including immunoassay, biosensor, bioseparation, targeted drug delivery, and environmental analysis have also been summarized.

## 2. Methods for Preparation of Magnetic Nanoparticles

Magnetic nanoparticles can be synthesized by physical, chemical and biological methods: (i) physical methods [35,36,37,38], such as gas-phase deposition and electron beam lithography; (ii) wet chemical preparation methods, such as sol−gel synthesis [39,40], oxidation method [41,42], chemical coprecipitation [43,44], hydrothermal reactions [45,46], flow injection synthesis [47], electrochemical method [48,49], aerosol/vapor phase method [50,51], sonochemical decomposition reactions [52,53], supercritical fluid method [54,55], synthesis using nanoreactors [56,57] and (iii) microbial methods [58,59,60].

As shown in Table 1, each of the above mentioned methods have their own advantages and disadvantages. Although physical methods are easy to perform, it is rather difficult to control the particle size with them. In the case of wet chemical preparation methods, some of them yield efficient control of the particle size by carefully adjusting the involved parameters, such as sol–gel method, chemical coprecipitation, hydrothermal method, flow injection method, electrochemical method, sonochemical decomposition method, supercritical fluid method and synthesis using nanoreactors. In particular, chemical coprecipitation technique of iron salts in aqueous medium might be the simplest and most efficient pathway to obtain iron magnetic nanoparticles. It has been demonstrated the particle size as well as the polydispersity of the nanoparticles could be tailored by changing the associated factors such as Fe^2^^+^/Fe^3^^+^ ratio [43], base (NaOH, ammonium hydroxide, and CH_3_NH_2_) [61], ionic strength (N(CH_3_)^4^^+^, CH_3_NH^3^^+^, NH^4^^+^, Na^+^, Li^+^ and K^+^) [62]. Some other apparently small factors also have an influence on the size of the nanoparticles [63]. For example, an increase of the mixing rate or temperature tends to decrease the particle size. Inlet of nitrogen into the reaction system not only protects against critical oxidation of the magnetite but also reduces the particle size when compared to methods without oxygen removal. Microbial methods ensure high yield, good reproducibility, and good scalability, as well as low cost, but the fementation process is rather time-consuming.

**Table 1:** Comparation between methods for synthesis of magnetic nanoparticles.

Methods		Advantages	Disadvantages
physical methods	gas-phase deposition	easy to perform	difficult to control the particle size
	electron beam lithography	well controlled inter-particle spacing	expensive and highly complex machines requiring
wet chemical preparation methods	sol−gel synthesis	precisely controlled in size, aspect ratio, and internal structure	weak bonding, low wear-resistance, high permeability
	oxidation method	uniform size and narrow size distribution	small-sized ferrite colloids
	chemical coprecipitation	simple and efficient	not suitable for the preparation of high pure, accurate stoichiometric phase
	hydrothermal reactions	easy to control particle size and shapes	high reaction temperature, high pressure
	flow injection synthesis	good reproducibility and high mixing homogeneity together with a precise control of the process	need continuous or segmented mixing of reagents under a laminar flow regime in a capillary reactor
	electrochemical method	easy to control particle size	reproducibility
	aerosol/vapor phase method	high yields	extremely high temperatures
	sonochemical decomposition reactions	narrow particle size distribution	mechanism not still understood
	supercritical fluid method	efficient control of the particle size, no organic solvents involved	critical pressure and temperature
	synthesis using nanoreactors	the possibility to precisely control the NP size	complex condition
microbial methods	microbial incubation	high yield, good reproducibility, and good scalability, low cost	time-consuming

To get the products with homogeneous composition and narrow size distribution, some purification procedure such as ultracentrifugation, size-exclusion chromatography are required.

## 3. Modification of Iron Magnetic Nanoparticles

Naked iron magnetic nanoparticles are generally unstable in strong acidic solutions and undergo leaching, which strongly limits the reusability and reduces the lifetime of such materials. The large ratio of surface area to volume brings in another limitation—aggregation of particles and thus a minimization in their surface energy ocurrs due to strong magnetic attractions between particles, limiting their dispersion in aqueous solutions and matrices. The exposition of the proteins/enzymes to such interfaces would result in the decrease or loss of the activity.

To overcome such limitations, various approaches are used to modify the surface via loading of other target chemicals or biological materials during or after the synthesis process. These techniques not only optimize the surface properties such as biocompatibility, dispersibility and biodegradability of iron magnetic nanoparticles, but also provide an environment for the transferring of hydrophobic iron oxide nanoparticles into a hydrophilic system.

Various materials have been involved in this process. Surfactants such as oleic acid, lauric acid, alkylsulphonic acids, and alkylphosphonic acids are common used molecules [64]. Several polymers such as polyethylene glycol (PEG), polyvinylpyrrolidone (PVP), poly(ethylene-co-vinyl acetate), poly(lactic-co-glycolic acid) (PLGA), and polyvinyl alcohol (PVA) have also been used as coating materials in aqueous suspension [65]. Moreover, natural dispersants including gelatin, dextran, polylactic acids, starch, albumin, liposomes, chitosan, ethyl cellulose have also been extensively employed for coating purpose in aqueous medium [66,67,68,69].

**Figure 1:**
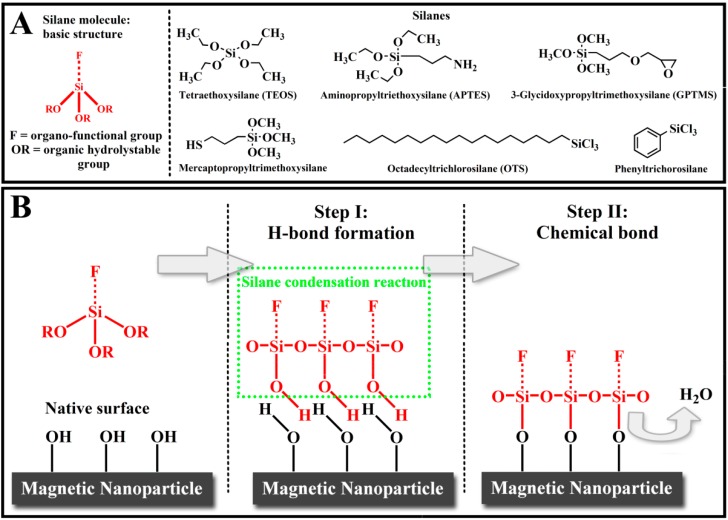
Chemical structures of mostly used silane precursors for surface functionalization and silanization reaction mechnism on magnetic nanoparticle. (**A**) Organo-functional silane molecule structures contain two different basic reactive groups: an organic hydrolyzable group (OR) and an organo-functional group (F); (**B**) Schematic of the condensation reaction between silane molecules and an oxide surface. The Si–OR bonds hydrolyze readily with water to form silanol Si–OH groups, which can then condense with each other to form polymeric structures with hydroxyl groups on the material surface.

Surface silanization is undoubtedly the most widely used technique to introduce surface functional groups on bare magnetic nanoparticles due to its characteristics such as satisfying responsivity, low cytotoxicity, high stability under acidic conditions, inertness to redox reactions and easy to perform surface chemical modification. Furthermore, the reaction can be either carried out in aqueous media or organic solvents at moderate temperatures, and no particular conditions or expensive equipment are required, therefore, it is considered as an ideal method for the protection of the inner magnetic core. The reaction mechanism is schematically shown in Figure 1 [70]. Silane molecules are firstly activated (hydrolysed) and then followed by the condensation reactions occurring between the Si–OH groups of the silanol and the OH groups of the surface. This leads to the formation of a stable bond on the surface.

Since the physical and chemical nature have decisive effects on the applicability of magnetic nanoparticles, comprehensive surface characterization techniques are utilized for a better understanding of the surface properties such as surface morphology, chemical composition and spatial distribution of the functional groups. Fundamental techniques [71,72,73,74,75,76] employed to investigate magnetic nanoparticles mainly include: Fourier transform infrared spectroscopy (FT-IR), scanning electron microscopy (SEM), transmission electron microscopy (TEM), X-ray photoelectron spectroscopy (XPS), atomic force microscopy (AFM), vibrating sample magnetometry (VSM), X-ray diffraction (XRD) analysis and thermal gravimetric analysis (TGA).

## 4. Immobilization Methods

Compared with the conventional immobilization methods, nanoparticle based immobilization can be characterized by two main features [77,78,79,80,81]: (i) the composition, size and morphology of particles can be conveniently tuned by changing the reaction conditions; (ii) uniform particles make it easy to perform the enzyme immobilization on a large scale without using surfactants and toxic reagents. The methods developed for protein immobilization onto iron magnetic nanoparticles mainly include physical immobilization, covalent conjugation and biologically-mediated specific interactions.

### 4.1. Physical Immobilization

Physical immobilization can be considered the simplest functionalization method employed in protein immobilization, as it may be easily carried out by just dipping the material into a solution containing the target biomolecules, and no additional coupling reagents, surface treatment and protein modification are required [82,83,84]. A good number of physically immobilized proteins based on iron magnetic nanoparticles have been developed. For example, glucose oxidase was immobilized onto Fe_3_O_4_ magnetic nanoparticles via physical method for water deoxygenation, and a 78% immobilization was obtained with specific activity of 640 U/g [85]. In another example, electrostatic interactions were employed to immobilize pectinase on negatively charged AOT-Fe_3_O_4_ nanoparticles, and a maximum specific activity (1.98 U/mg enzyme) of immobilized pectinase and maximum enzyme loading of 610.5 mg enzyme/g support was attained and the activity loss of immobilized protein was only 10%–20% after six cycles [86]. Although physical immobilization is simple and mild, this method generally involves comparatively weak interactions such as electrostatic interactions [87,88], hydrogen bonds [89], van der Waals forces [90,91,92], and hydrophobic interactions [93], and the binding stability of adsorbed species is highly affected by environmental conditions (pH, temperature, ionic strength and biomolecule concentration). Therefore, proteins immobilized using this method tend to break away from the support, and thus lead to the loss of activity and contamination of the reaction media, which would affect the robustness and recyclability of the immobilized proteins, particularly for those constructed for analytical and detective use. Furthermore, protein adsorption directly onto surfaces often leads to the denaturation of proteins and losses in protein activity resulting from conformational changes caused by steric interaction.

### 4.2. Covalent Conjugation

Covalent immobilization is particularly attractive, as it could be carefully regulated with specific functional groups to bind to proteins. Several immobilization protocols using covalent binding have already been developed and employed in protein immobilization [94,95,96,97].

Coupling agents such as glutaraldehyde (GA) [98,99,100,101,102] and 1-ethyl-3-(3-dimethylaminopropyl) carbodiimide hydrochloride (EDC) [103,104,105,106] are often utilized to covalently cross-link the modified magnetic nanoparticles and proteins because their functional groups (e.g., aldehyde group) can interact with both functional groups of the modified magnetic nanoparticle and proteins. For example, glucose oxidase (GOD) was immobilized on CoFe_2_O_4_/SiO_2_ NPs via cross-linking with glutaraldehyde. After immobilization, the GOD exhibited improved thermal, storage and operation stability. The immobilized GOD still maintained 80% of its initial activity. After storage at 4 °C for 28 days, the immobilized and free enzyme retained 87% and 40% of initial activity, respectively [98]. In another example, the Fe_3_O_4_–chitosan nanoparticles are used for the covalent immobilization of lipase from *Candida rugosa* using *N*-(3-dimethylaminopropyl)-*N*′-ethylcarbodiimide (EDC) and *N*-hydroxysuccinimide (NHS) as coupling agents. The optimum immobilization conditions were immobilization time 2.14 h, pH 6.37, and enzyme/support ratio 0.73 (w/w); the highest activity obtained was 20 U/g Fe_3_O_4_–chitosan. The immobilized lipase retains over 83% of its initial activity after twenty repeated cycles [103]. However, in many cases, the presence of coupling agents could cause a conformational change of proteins, resulting in a decrease of enzyme activity. For example, the immobilized enzymes retained 15%–23% of the native glucose oxidase. Recycling stability studies showed approximately 20% of activity loss for the glucose oxidase-magnetic nanoparticle bioconjugate [107].

Several other enzymes have been successfully immobilized onto iron magnetic nanoparticles using this covalent method, e.g., lipase [108], *Candida rugosa* lipase [109], horseradish peroxidase [110,111,112,113,114], pectinase [115,116], trypsin [117,118,119,120], α-chymotrypsin [121,122,123,124,125,126,127], cyclic adenosine monophosphate-dependent protein kinase [128], glucose oxidase [129,130,131,132,133,134,135], hexaarginine-tagged esterase [136], porcine pancreas lipase papain [137], *Saccharomyces cerevisiae* alcohol dehydrogenase [138], chitosanase [139,140], triacylglycerol lipase [141], laccase (a copper oxidoreductase from a fungal source) [142,143,144], epoxide hydrolase [145,146], *etc.*

The main problem in the practical use of covalent immobilization is non-specificity, and purified proteins are generally involved in covalent immobilization. However, purification of proteins in large scale is really time-consuming and resource intensive, and the impurities rather than the target protein present in the mixture might also have strong tendency to bind to the support which would great affect its activity. Another aspect associated with covalent immobilization is once the activity of immobilized protein/enzyme decays, the support must be discarded together.

### 4.3. Biologically Mediated Specific Interaction

Many of the applications demonstrated thus far have relied on protein attachment methods that result in non-site-specific immobilization, either through adsorption or covalent attachment. However, such non-specificity greatly limits their application. Site-specific immobilization methods based on biological reactions have offered a novel route to solve the problem of selectivity, which could be achieved by the formation of bonds between the active groups on the supports and specific residues on the protein. By proper modification of the supports and protein engineering, such attachments could be strategically realized under mild conditions, which have greatly reduced the risk of protein degradation or denaturation.

**Scheme 1:**
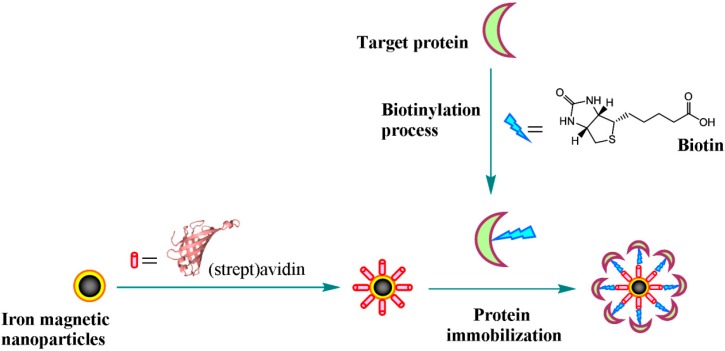
Schematic illustration of protein immobilization onto iron magnetic nanoparticles using (strept)avidin–biotin technology.

The (strept)avidin–biotin technology probably ranks among the most extensively researched and frequently used protein-mediated immobilization technique that relies on biologically mediated immobilization methods [147] (Scheme 1). The biotin and (strept)avidin couple not only has a high binding affinity (*K*_d_ ≈ 10^−15^ M), but also exhibits high specificity, hence it finds potential application in protein immobilization. The four biotin binding sites positioned in pairs on a stretavidin’s opposite domains can serve as a bridge between the immobilized biotinylated moiety and the target nanoparticles. The proteins functionalized with (strept)avidin are unusually stable to extremes such as heat, denaturants, pH, and proteolysis, indicating the binding is essentially irreversible. For instance, Nidumolu’s group reported the synthesis of streptavidin-functionalized magnetic nanoparticles and investigation of the binding to biotinylated SAMs on gold and glass for biological recognition applications [148]. The whole process includes three steps. Firstly, magnetic nanoparticles was prepared using traditional chemical coprecipitation method, which were then modified with the protein FITC-labeled streptavidin using carbodiimide activation. Secondly, gold and glass surfaces were functionalized with biotin SAMs by applying biotin-HPDP onto gold coated glass slides. Thirdly, the functionalized nanoparticles specifically bind to the biotinylated gold surfaces.

Even though the selectivity has contributed greatly to the popularity of this method as a means of protein immobilization, the protein of interest must first be labeled with biotin if site-selective attachment is desired.

## 5. Aplications of Magnetic Nanoparticles Immobilized Proteins

Magnetic nanoparticles appear to be very promising for their applications in bioseparation, medical science, and environmental analysis. In this section, these applications will be discussed briefly.

### 5.1. Bioseparation

Affinity chromatography is one of the most efficient strategies for the efficient purification of recombinant protein due to the affinity interactions between metal ions (e.g., Ni^2+^) and polyhistidine affinity tag (His tag). Several functionalized MNPs have been developed for the specific separation of different proteins (Table 2).

**Table 2:** Examples of proteins separated by modified magnetic techniques [149].

Protein	Magnetic carrier	Ligand	Elution method	Reference number
Lysozyme	Fe_3_O_4_ @ PEG @ CM-CTS	-COOH	PBS containing NaCl	[150]
	Fe_3_O_4_ @ SiO_2_ @ GPS @ Tris	Tris	n/a	[151]
	Fe_3_O_4_ @ PAA	-COOH	Phosphate buffer containing NaSCN	[152]
	Magnetic PHEMA beads @	Cibacron Blue F3GA	Tris/HCl buffer containing NaCl	[153]
SOD	Fe_3_O_4_ @ IDA@Cu^2^^+^	IDA@Cu^2^^+^	Potassium phosphate in the presence of NH_4_Cl	[154]
Lipase	Fe_3_O_4_ @ PAA	-COOH	Phosphate buffer (pH 9)	[155]
His-tag proteins	Fe_3_O_4_ @ PMIDA-Ni^2^^+^	PMIDA-Ni^2^^+^	Sodium phosphate, NaCl and imidazole	[156]
Lactoferrin	Fe_3_O_4_ @ PGMA-EA @ heparin	Heparin	NaCl	[157]
BHb	Fe_3_O_4_ @ SiO_2_ @ GPS @ IDA-Zn^2^^+^	DA-Zn^2^^+^	n/a	[158]
Antibody	Fe_3_O_4_ @ cellulose @ protein A	Protein A	n/a	[159]

n/a Not available or not possible to determine from the information published. CM-CTS = carboxymethyl chitosan, GPS = 3-glycidoxypropyltrimethoxysilane, PEG = polyethylene glycol, Tris = tris(hydroxymethyl)-amino-methane, PAA = polyacrylic acid, SOD = superoxide dismutase, IDA = iminodiacetic acid, PMIDA = N-phosphonomethyl iminodiacetic acid, EA = ethanediamine, PGMA = polyglycidyl methacrylate, BHb = bovine haemoglobin.

Once captured, the target proteins, in most cases, could be rapidly and completely eluted by using proper elution methods, generally, buffer solution with different pH or ion strength. The change of pH can change the surface electric charges of the protein and the support, and thus decrease the interaction between the protein and the ligand-immobilized magnetic nanoparticles. The increase of ion strength, usually realized by the addition of salts such as Na^+^, could reduce electrostatic reaction between the protein and the ligand-immobilized magnetic nanoparticles. Therefore, these two parameters are taken into account to select proper buffer for protein elution from the ligand-immobilized magnetic nanoparticles. However, the use of excessively high (>10) or low (<4) pH for protein elution might significantly impact the bioactivity of proteins.

Imidazole, as an efficient and gentle regent, is very suitable for the elution of target proteins for metal ion chelated magnetic nanoparticles. Ma, Liu, Guan, and Liu [158,160] used imidazole solution as eluent for the elution of the adsorbed bovine serum albumin (BSA) from Cu^2^^+^ chelated magnetic nanoparticles and for the elution of BHb and BSA from Zn^2^^+^ chelated magnetic nanoparticles. Sahu *et al.* [156] used imidazole solution to elute His-tagged recombinant proteins for Ni^2^^+^ chelated magnetic nanoparticles. These metal ion chelated magnetic nanoparticles were usually regenerated by using EDTA to strip the adsorbed protein and metal ion and then reloading with metal ion for repeated use.

In comparison, several adsorbed proteins are difficult to elute from ligand-immobilized magnetic nanoparticles, which may be ascribed to the complex interactions between the ligands and target proteins or other factors. Zheng *et al.* prepared magnetic core-shell Fe_3_O_4_@SiO_2_@poly(styrene-alt-maleic anhydride) spheres for the separation of His-tagged proteins from cell lysates [161]. The enrichment capacity of this magnetic polymer was four times greater than that by Fe_3_O_4_@SiO_2_/Ni-NTA. Also, Bruening *et al.* synthesized polymer-brush-modified MNPs functionalized with nitrilotriacetate-Ni^2+^ by ATRP to capture histagged proteins selectively from cell extracts [162]. The polymer brushes can dramatically increase the adsorption capacity for his-tagged ubiquitin and afford high protein recoveries. A novel three-component microsphere (Fe_3_O_4_@SiO_2_@NiAl-LDH), which possesses large surface area and uniform mesochannels, was prepared by Wei *et al.* via an *in situ* growth method [163]. The Fe_3_O_4_@SiO_2_@NiAl-LDH microspheres prepared showed a binding capacity of 239 µg/mg beads for the adsorption of His-tagged green fluorescent protein.

### 5.2. Medical Science

#### 5.2.1. Targeted Drug Delivery

Drug delivery systems based on magnetic nanoparticle have major advantages over the conventional, non-targeted methods of drug delivery. In conventional drug delivery systems, the poor specificity for the target site and reduced drug diffusion through biological barriers often lead to a suboptimal pharmacological activity and high incidence of adverse effects. In contrast, magnetic nanoparticles are promising stimuli-sensitive drug carriers due to the better retention ability than those of small molecules. An applied extracorporeal magnetic field can concentrate these nanoparticles at the desired site to receive enough drug efficiency and an improved therapeutic activity, ensuring sustained but complete release of the immobilized drugs. In particular, nanoparticles in the range of 20–400 nm in diameter show tumor-selective integration known as the enhanced permeability and retention (EPR) effect.

Drugs whose therapeutic doses are low, but having strong electrostatic affinity toward MNPs, can be loaded directly by adsorption onto the nanoparticle surface. However, the drug loading just by surface adsorption is generally insufficient to reach the therapeutic drug concentration necessary at the target site. Thus, a core-shell structure including a magnetic core and a biodegradable organic or inorganic shell is usually employed in magnetic drug-delivery systems [164] to achieve sufficient drug payload. Once the drug carrier is concentrated at the target, the drug can be released either via enzymatic activity or changes in physiological conditions such as pH, osmolality, or temperature, and may be internalized by the endothelial cells of the target tissue or be taken up by the tumour cells. For example, Jana *et al.* reported a targeted delivery of serratiopeptidase enzyme immobilized on magnetic nanoparticles of Fe_3_O_4_. This magnetic drug-delivery system increased the delivery of drug through the membrane in *in vitro* study and enhanced the anti-inflammatory effect on in rats in *in vivo* study [165].

#### 5.2.2. Bisensor

Iron magnetic nanoparticles coated with other materials, such as a fluorescent ones, a metal, silica, or a polymer, are used as bioanalytical sensors. For example, Fe/Fe_2_O_3_ core/shell nanoparticles attached with bioactive proteins such as cytochrome P450, myoglobin (Mb), and hemoglobin (Hb) are selectively used for the detection of damaged DNA [166,167]. Magnetic nanoparticles have been also applying in glucose biosensors which use immobilized oxidase for the conversion of the target analytes into electrochemically detectable products for the determination of glucose in foodstuff samples [149]. An iron oxide nanoparticles-chitosan composite based glucose biosensor was developed by Kaushik *et al.* [168]. This biosensor response time of 5 s, linearity as 0.1–4 mg/mL of glucose, sensitivity as 9.3 × 10^−2^ mA/(mg mL cm^2^) and shelf life of about 8 weeks under refrigerated conditions. The use of iron oxide nanoparticles-chitosan composite enhanced the enzymatic activity and stability of glucose oxidase, which results in high sensitivity and long shelf life.

#### 5.2.3. Bioimaging

Magnetic resonance imaging (MRI) is a commonly used non-invasive medical imaging technique in clinical medicine to visualize the structure and function of tissues [169,170], which is based on the behavior, alignment and interaction of protons in the presence of an applied magnetic field. Due to prorpeties such as biocompatibility, high saturation magnetization, superparamagetism, and low toxicity, proteins immobilized on iron magnetic nanoparticles have been successfully applied in MRI imaging [171]. Human holo-transferrin conjugated to iron oxide nanoparticles showed that increases in receptor levels at the cell surface can cause considerable changes in MRI signals. These superparamagnetic iron oxide nanoparticles are relatively non-toxic when administered intravenously, and similar preparations are in clinical use.

### 5.3. Food Analysis

Food safety has attracted increasing attention by consumers, governments, and manufacturers. Magnetic nanoparticles are usually integrated with detection techniques for food analysis by two ways: electrode modifier and sample pre-concentrator. For example, a protein A coated magnetic microparticles-based enzyme-linked immunosorbent assay method was developed for the detection of Ara h3/4 peanut allergen in foods, which had a detection limit as low as 0.2 mg/kg and achieved excellent precision and repeatability [172].

## 6. Concluding Remarks and Prospects

Nanoparticles have emerged as versatile tools for generating excellent supports for enzyme stabilization due to their small size and large surface area. By proper surface modification, various iron magnetic nanoparticles have been synthesized and successfully utilized for protein/enzyme immobilization, which have already displayed promising effects in practical applications. We have summarized in this review the applications of iron magnetic nanoparticles in enzyme immobilization, protein separation/purification, medical science and food analysis. The immobilized proteins/enzymes generally show better stability towards pH and heat than the free ones and can be recovered and reused multiple times. However, the activity of some enzymes decreases to some extent after immobilization, which indicates that more efforts are still required to explore the immobilization techniques.
